# Human *cytochrome P450 2B6* genetic variability in Botswana: a case of haplotype diversity and convergent phenotypes

**DOI:** 10.1038/s41598-018-23350-1

**Published:** 2018-03-20

**Authors:** Leabaneng Tawe, Thato Motshoge, Pleasure Ramatlho, Naledi Mutukwa, Charles Waithaka Muthoga, Ghyslaine Bruna Djeunang Dongho, Axel Martinelli, Elias Peloewetse, Gianluca Russo, Isaac Kweku Quaye, Giacomo Maria Paganotti

**Affiliations:** 1 University of Botswana, Department of Medical Laboratory Sciences, Gaborone, Botswana; 2 Botswana-University of Pennsylvania Partnership, Gaborone, Botswana; 3 University of Botswana, Department of Biological Sciences, Gaborone, Botswana; 4 University of Botswana, Department of Pathology, Gaborone, Botswana; 5 Sapienza University of Rome, Department of Infectious Diseases and Public Health, Rome, Italy; 6 Evangelical University of Cameroon, Department of Biomedical Sciences, Bandjoun, Cameroon; 7 Global Institution for Collaborative Research and Education, Hokkaido University, Sapporo, Japan; 8 King Abdullah University of Science and Technology, Biological and Environmental Sciences and Engineering Division, Thuwal, Saudi Arabia; 9 University of Namibia, Department of Biochemistry, Windhoek, Namibia; 10 University of Pennsylvania, Perelman School of Medicine, Philadelphia, PA USA; 11 University of Botswana, Department of Biomedical Sciences, Gaborone, Botswana; 12 Present Address: Sub-Saharan African Network for TB/HIV Research Excellence at Botswana-Harvard Partnership, Gaborone, Botswana

## Abstract

Identification of inter-individual variability for drug metabolism through cytochrome P450 2B6 (CYP2B6) enzyme is important for understanding the differences in clinical responses to malaria and HIV. This study evaluates the distribution of *CYP2B6* alleles, haplotypes and inferred metabolic phenotypes among subjects with different ethnicity in Botswana. A total of 570 subjects were analyzed for *CYP2B6* polymorphisms at position 516 G > T (rs3745274), 785 A > G (rs2279343) and 983 T > C (rs28399499). Samples were collected in three districts of Botswana where the population belongs to Bantu (Serowe/Palapye and Chobe) and San-related (Ghanzi) ethnicity. The three districts showed different haplotype composition according to the ethnic background but similar metabolic inferred phenotypes, with 59.12%, 34.56%, 2.10% and 4.21% of the subjects having, respectively, an extensive, intermediate, slow and rapid metabolic profile. The results hint at the possibility of a convergent adaptation of detoxifying metabolic phenotypes despite a different haplotype structure due to the different genetic background. The main implication is that, while there is substantial homogeneity of metabolic inferred phenotypes among the country, the response to drugs metabolized via CYP2B6 could be individually associated to an increased risk of treatment failure and toxicity. These are important facts since Botswana is facing malaria elimination and a very high HIV prevalence.

## Introduction

The human cytochrome P450 2B6 enzyme (CYP2B6) plays a pivotal role in the metabolism of different drugs used for malaria treatment (artemisinin derivatives such as artesunate, β-artemether and artemether) and for HIV life-long therapy (non-nucleoside reverse-transcriptase inhibitors such as efavirenz and nevirapine). CYP2B6 is a highly polymorphic enzyme that affects the therapeutic response including drug interactions in individuals^1,2^. Importantly, African populations show a high degree of variation in the *CYP2B6* gene^3^. In Botswana, interventions towards the elimination of *Plasmodium falciparum* malaria have been intensified and HIV has been reported at a frequency of 18.5%^4^. So, a deeper knowledge of the genetic variability of *CYP2B6* in the population of Botswana is necessary to improve the efficacy of ongoing fight against malaria and HIV.

Currently, the first line treatment for uncomplicated malaria in Botswana is a fixed-dose combination of artemether (AM) and lumefantrine (LU)^5^. After oral administration, AM is rapidly absorbed and metabolized to dihydroartemisinin (DHA) through demethylation, mainly via CYP2B6 enzymes^6–8^. DHA has a higher antimalarial activity than AM, and it is inactivated primarily by glucuronidation^7,9^.

Efavirenz (EFV) and nevirapine (NVP) are the most prescribed drugs in anti-HIV combination treatment in resource-limited countries^10^. There is a considerable literature on EFV and NVP pharmacogenetics and currently it has been proposed that personalizing the dosage of these anti-HIV drugs according to the *CYP2B6* genotype of the patient will be of therapeutic benefit^11^.

The known single nucleotide polymorphisms (SNPs) within the *CYP2B6* locus influencing AM and EFV/NVP plasma exposure are: 516 G > T (rs3745274), 785 A > G (rs2279343) and 983 T > C (rs28399499). The polymorphisms at position 516 and 983 confer a slow metabolic phenotype, leading to higher drug plasma exposure and increased toxicity risk. The variation at position 785 confers rapid drug metabolism, leading to lower drug plasma level and potentially poorer therapeutic outcome^10^. The above-mentioned SNPs, in combination or individually, may lead to the following *CYP2B6* alleles: *4 (785 A > G only), *6 (516 G > T and 785 A > G), *9 (516 G > T only), *16 (785 A > G and 983 T > C), *18 (983 T > C only) (http://cypalleles.ki.se). The metabolic status for a given drug is defined as slow, intermediate, extensive or ultra-rapid as a result of the individual’s genetic make up^12^. Furthermore, in the anti-infectious therapy context, the therapeutic outcome based on the individual metabolic status for a given drug depends also on the length and frequency of therapy. For example, in the context of the short course uncomplicated malaria treatment, a slow AM metabolism induces a lower biotransformation rate to the most active metabolite DHA^7^ leading to a lower efficacy and possibly a longer tail during which malaria parasites are exposed to sub-inhibitory drug concentrations, inducing an increased permanence in the selective window for drug resistance^13,14^. Conversely, in the context of chronic EFV- or NVP-based antiretroviral combination therapy (ARTc), a slow metabolic profile reduces drug clearance leading to higher drug plasma exposure and consequent higher toxicity risk. This may cause poorer compliance by the patient with resultant emergence of viral resistance possibly related to suboptimal drug exposure^10,15^. Concerning the influence of rapid metabolism phenotypes on antimalarial and antiretroviral therapy, information is lacking^10^.

Another important aspect is that malaria treatment in sub-Saharan Africa may frequently overlap EFV- or NVP-based ARTc leading to possible drug-drug interactions. The co-administration of EFV or NVP and AM-LU was associated with low plasma concentration of AM and DHA, whereas LU concentration was reduced by EFV co-administration^16,17^. A recent study, looking at HIV-infected patients affected by malaria in Tanzania, has shown that the slow metaboliser genotype CYP2B6 *6/*6 leads to high EFV plasma concentration which are significantly correlated with low LU plasma concentrations and a high rate of recurrent parasitemia^18^, increasing the risk of the appearance of drug-resistant malaria parasites.

Finally, Botswana (together with other Southern African countries) is also home to individuals with unique southern African KhoeSan ancestry. The KhoeSan populations are the earliest known indigenous inhabitants of southern Africa and are distinguished by their unique phenotype(s), genetic divergence, click languages, and hunter-gatherer subsistence strategy compared to other African populations^19–21^. During evolution, hunter-gatherer practices exposed populations to several xenobiotics which sometimes imposed serious health and environmental risks, leading to the selection of specific mutations linked to an efficient detoxification. For example, significant differences in prevalence of acetylation phenotypes are found between hunter-gatherer and food-producing populations, both in sub-Saharan Africa and worldwide, and between agriculturalists and pastoralists in Central Asia^22^. In Africa, comparative studies on N-acetyl transferase (*NAT2*) haplotype frequencies and acetylation status inference revealed that the hunter-gatherer populations (San, but also Pygmies) are mainly composed of fast and intermediate acetylators^23^, in clear contrast with most agriculturalist populations (such as Bantu). A recent report showed differences in the allele frequency for CYP2C8*2 among San and Bantu-related communities in Botswana^24^. These general observations highlight the need for a detailed pharmacogenetic characterization of populations of pure or admixed KhoeSan ancestry in Botswana to accommodate individual/population genetic make-up and expedite optimum treatment strategies against important infections such as malaria and HIV infection.

**Figure 1:**
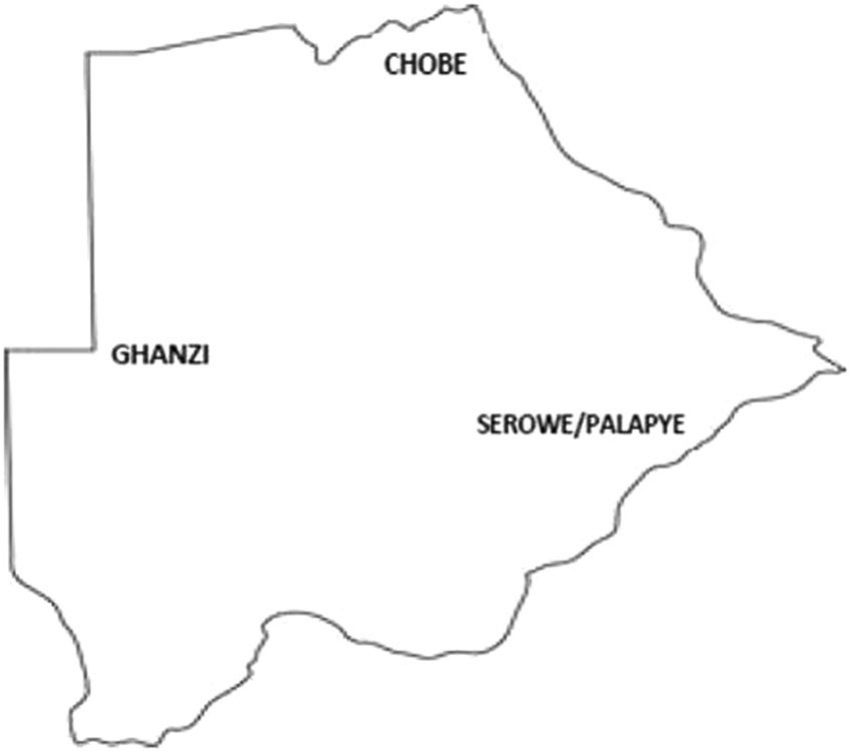
Map of Botswana.

In this study we aim to: *i*) define the *CYP2B6* haplotype status in Botswana via the three most important functional SNPs known to affect the enzyme activity; *ii*) examine the relationship, if any, of the haplotypes within ethnic groups; *iii*) apply the “metabolic score” in the study of inferred metabolic phenotypes among the populations. We selected three sites where the ethnic composition is different (San-related population in the Ghanzi health district and Bantu-related populations in the Chobe and Serowe/Palapye health districts, respectively) (see Fig. 1). This work is part of the ongoing screening activities for antimalarial drugs efficacy and safety study in Botswana unto malaria elimination by 2020, but it also provides useful information on antiretroviral drug metabolism in the population, making this research of public health relevance for the country.

## Results

Out of 609 samples we obtained genotypic data for all the three polymorphisms from 570 samples (93.60%). The genotype and allele frequency for 516 G > T, 785 A > G and 983 T > C in the three selected districts are shown in Table 1 with statistical comparisons.

**Table 1:** Genotype and allele frequencies for *CYP2B6* 516-785-983 SNPs and comparisons among the groups.

District	516 G > T				HWE (*P*)	785 A > G				HWE (*P*)	983 T > C				HWE (*P*)	Total
	GG (%)	GT (%)	TT (%)	*f*(T)		AA (%)	AG (%)	GG (%)	*f*(G)		TT (%)	TC (%)	CC (%)	*f*(C)		
Serowe/Palapye (S/P)	104 (40.62)	106 (41.41)	46 (17.97)	38.67	Het-d (*0*.*038*)	108 (42.19)	109 (42.58)	39 (15.23)	36.38	ok	183 (71.48)	73 (28.52)	0 (0.00)	14.26	Het-e (<*0*.*001*)	256
Chobe (Ch)	46 (29.49)	74 (47.44)	36 (23.07)	46.79	ok	49 (31.41)	80 (51.28)	27 (17.31)	42.95	ok	107 (68.59)	47 (30.13)	2 (1.28)	16.35	ok (*0*.*061*)	156
[S/P + Ch]	150 (36.41)	180 (43.69)	82 (19.90)	41.75	Het-d (*0*.*025*)	157 (38.11)	189 (45.87)	66 (16.02)	38.96	ok	290 (70.39)	120 (29.13)	2 (0.48)	15.05	Het-e (<*0*.*001*)	412
Ghanzi (GH)	81 (51.26)	64 (40.51)	13 (8.23)	28.48	ok	107 (67.72)	47 (29.75)	4 (2.53)	17.41	ok	128 (81.01)	30 (18.99)	0 (0.00)	9.49	ok	158
All	231 (40.53)	244 (42.81)	95 (16.66)	38.07	Het-d (*0*.*021*)	264 (46.32)	236 (41.40)	70 (12.28)	32.98	ok	418 (73.33)	150 (26.31)	2 (0.36)	13.51	Het-e (<*0*.*001*)	570
**Comparison: Yates-corrected chi-square value (with** ***P*** **), df = 1; OR (95% CI)**																
S/P vs Ch	4.93 (*0*.*022*)			1.39 (1.04–1.87)		3.1 (*0*.*067*)			1.31 (0.97–1.76)		0.51 (*0*.*416*)			1.17 (0.78–1.76)		
S/P vs GH	8.50 (*0*.*003*)			0.63 (0.46–0.86)		33.61 (≪*0*.*001*)			0.37 (0.26–0.52		3.65 (*0*.*044*)			0.63 (0.39–10.1)		
Ch vs GH	21.67 (≪*0*.*001*)			0.45 (0.32–0.64		47.48 (≪*0*.*001*)			0.28 (0.19–0.41)		5.97 (*0*.*010*)			0.54 (0.32–0.89)		
[S/P + Ch] vs GH	16.49 (≪*0*.*001*)			0.55 (0.41–0.74)		47.02 (≪*0*.*001*)			0.33 (0.24–0.46)		5.57(*0*.*014*)			0.59 (0.38–0.92)		

HWE: Hardy-Weinberg equilibrium test (with *P*, when significant). Het-d: significant heterozygous defect; Het-e: significant heterozygous eccess.

Comparisons were made among districts of different ethnic composition (pairwise or combined) for each SNP.

### Hardy-Weinberg Equilibrium

Hardy-Weinberg equilibrium analysis showed that *CYP2B6*-516 displayed significant deviations from Hardy–Weinberg equilibrium in samples from Serowe/Palapye district due to a significant heterozygous defect (Wright’s F = 0.13), with the same sample having a significant *CYP2B6-*983 heterozygous excess (Wright’s F** = **−0.17). Genotypes in samples from Chobe and Ghanzi districts were in Hardy-Weinberg equilibrium. *CYP2B6-*785 genotypes were in equilibrium in all three districts analysed. Regarding the only two samples showing a rare CC genotype for 983 T > C polymorphism^25,26^, we sequenced them and confirmed the result.

### Linkage Disequilibrium analysis

A significant linkage disequilibrium (LD) was found with Arlequin between all pairwise comparisons of the three polymorphic loci when samples were considered as a single population (n = 570). Chi-square test values ranged from 36.07 (*P* < 0.00001, 1 df) for *CYP2B6*-516 vs *CYP2B6*-983, to 443.55 (*P* < 0.00001, 1 df) for *CYP2B6*-516 vs *CYP2B6*-785, to 43.93 (*P* < 0.00001, 1 df) for *CYP2B6*-983 vs *CYP2B6*-785. When analysing the samples separately by district of origin, we found a significant LD in all districts for all pairwise comparisons between the three loci (Table 2). In general, LD between *CYP2B6*-516 and *CYP2B6*-785 was strongest, while LD between *CYP2B6*-983 and the other two loci was weakest but still highly significant.

**Table 2:** Pairwise LD analysis for the three polymorphic loci. Chi-square values and *P*-value for LD analysis were obtained using Arlequin. Significance is assumed for *P* < 0.05.

District	Comparison	Chi-square	*P*-value (1 df)
Serowe/Palapye	516 vs 785	198.19	<0.00001
Serowe/Palapye	516 vs 983	22.54	<0.00001
Serowe/Palapye	785 vs 983	25.36	<0.00001
Chobe	516 vs 785	147.27	<0.00001
Chobe	516 vs 983	5.94	0.014760
Chobe	785 vs 983	8.32	0.003920
Ghanzi	516 vs 785	81.29	<0.00001
Ghanzi	516 vs 983	4.79	0.028660
Ghanzi	785 vs 983	4.88	0.027110

### Haplotype frequency estimation

Haplotype frequencies were estimated using Arlequin (Table 3). For the combined samples, the GAT haplotype was the most common and the GGC haplotype the rarest. Among the identified haplotypes, two not yet categorized were found, TGC and TAC, indicated as *6 + *18 and *9 + *18, respectively (Table 3). When districts were considered separately, GAT remained the dominant haplotype while GGC remained the rarest. However, differences in haplotype frequencies could be observed between districts. While there was no statistical difference in the population structure between the Serowe/Palapye and Chobe districts (Population pairwise FSTs test: *P* = 0.06 +/− 0.024, 110 permutations), there was a significant difference when both districts were separately compared to the Ghanzi district (Population pairwise FSTs test: *P* < 0.00001, 110 permutations for both comparisons). When the Serowe/Palapye and Chobe districts were combined and then compared to the Ghanzi district, the populations were still significantly different (Population pairwise FSTs test: *P* < 0.00001, 110 permutations). In particular the Ghanzi district displayed in particular a higher frequency (as estimated by ML) of the GAT (65.55%), TAT (10.98%) and TAC (1.53%) haplotypes and a lower frequency of the TGT (12.79%) and TGC (3.18%) haplotypes (Table 3). These data indicated a distinct population structure in terms of the *CYP2B6* alleles in the Ghanzi district compared to both the Serowe/Palapye and Chobe districts.

**Table 3:** Haplotype frequencies by district and for all the samples combined. Maximum-likelihood (ML) haplotype frequencies were calculated using the EM algorithm in Arlequin.

Haplotype	*CYP2B6* allele	Absolute and (ML) frequencies			
		S/P	Ch	Gh	All
GAT	*1	271 (0.52)	145 (0.45)	210 (0.66)	626 (0.54)
TGT	*6	113 (0.22)	90 (0.30)	38 (0.13)	241 (0.22)
TGC	*6/*18	49 (0.09)	35 (0.10)	13 (0.03)	97 (0.08)
TAT	*9	33 (0.07)	19 (0.06)	35 (0.11)	87 (0.08)
GAC	*18	18 (0.04)	12 (0.05)	12 (0.04)	42 (0.04)
GGT	*4	22 (0.05)	7 (0.02)	3 (0.01)	32 (0.03)
TAC	*9/*18	3 (0.01)	2 (0.01)	4 (0.01)	9 (0.01)
GGC	*16	3 (0.01)	2 (0.01)	1 (0.00)	6 (0.01)

### Neutrality tests

For each sample, the most likely gametic phases (as determined by Arlequin) were selected to perform neutrality tests. The results of the Tajima’s D tests indicated positive values above 2 for the Serowe and Chobe populations with significant *P*-values and 1.26 for the Ghanzi samples with a non-significant *P*-value. The Chobe and Serowe combined population dataset (*i*.*e*. Bantu) also yielded a statistically significant D value above two (Table 4). The significantly positive Tajima’s D scores among the Bantu groups could either indicate the presence of balancing selection, migration or a sudden population contraction, or even indicate positive selection on pre-existing genetic variation^27^.

**Table 4:** Results of the Tajima’s D neutrality test. Tests were performed with DnaSP v6. P-values are provided in brackets.

Population	Serowe/Palapye (S/P)	Chobe (Ch)	[S/P + Ch]	Ghanzi (Gh)
Tajima’s D (*P*-value)	2.39583 (<0.05)	2.50852 (<0.05)	2.63745 (<0.05)	1.25562 (>0.1)

### Metabolic score

**Figure 2:**
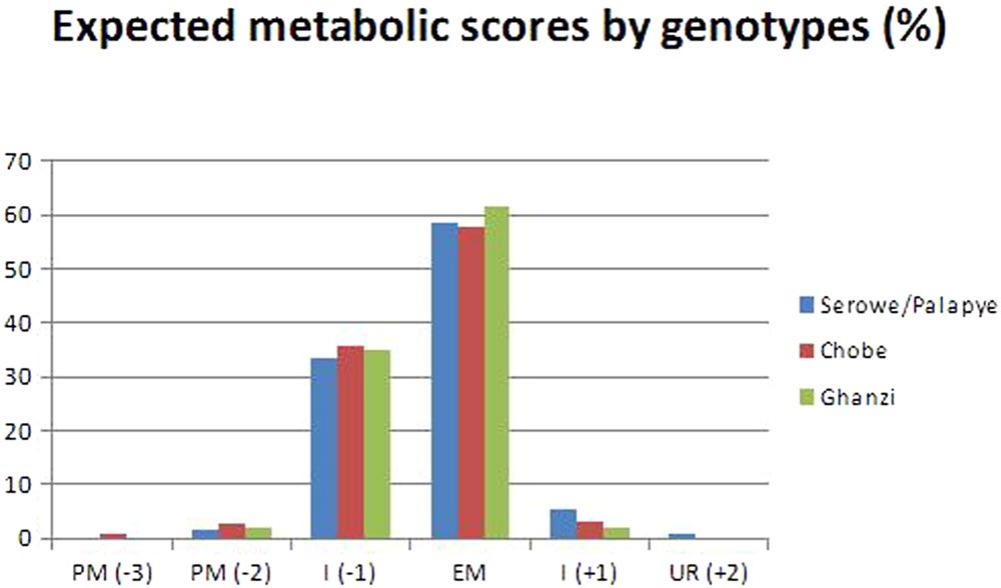
Distribution of the inferred metabolic scores by districts. PM = poor metabolisers; I = intermediate metabolisers (either with delayed or increased metabolism); EM = extensive or ‘normal’ metabolisers; UR = ultra-rapid metabolisers.

The expected metabolic scores were analysed among the three populations (Table 5 and Fig. 2).

**Table 5:** Distribution of the inferred metabolic phenotype and scores by district, and Kolmogorv-Smirnov (K-S) *D* statistics with associated *P* values to test the normal distribution of data.

District	PM (−3)	Expected metabolic phenotypes (with scores)						
		PM (−2)	I (−1)	EM (0)	I (+1)	UR (+2)	Total	K-S *D* statistic (*P*)
Serowe/Palapye	0	4	86	150	14	2	256	0.34 (*n*.*s*.)
Chobe	1*	4*	56	90	5	0	156	0.38 (*n*.*s*.)
Ghanzi	0	3	55	97	3	0	158	0.38 (*n*.*s*.)
Total	1	11	197	337	22	2	570	0.36 (*n*.*s*.)

Score attribution was made according to: 516 TT = −2; 516 GT = −1; 516 GG = 0; 785 GG = +2; 785 AG = +1; 785 AA = 0; 983 CC = −2; 983 TC = −1; 983 TT = 0. We performed a summation for each genotype (516, 983 and 785) per sample to obtain the “metabolic score”. PM: poor metabolisers; I: intermediate metabolisers; EM: extensive metabolisers; UR: ultra-rapid metabolisers. (*two 983CC genotypes were found in Chobe).

The Kolmogorov-Smirnov test did not find any statistically significant divergence from normal distribution for the metabolic scores in each of the three sub-samples (Table 5). The Bartlett test for homogeneity of variances showed that the three variances were not statistically different from each other (Bartlett’s chi square = 3.32, df = 2, *P* = 0.19). The chi-square statistic in the comparison of the absolute frequencies distribution of the metabolic scores among all districts was 9.43 with the associated *P*-value being 0.49, indicating that the distributions of metabolic scores in the three groups were comparable. The *CYP2B6* inferred phenotypes associated with a reduced metabolism were found in 36.67% (n = 209/570) of the overall population studied, mainly intermediate (94.25%, n = 197/209) rather than poor metabolizer (5.75%, n = 12/209). Phenotypes associated with increased metabolism were found in 4.21% (n = 24/570) of the population studied, with only 2 individuals showing an inferred ultra-rapid metabolic phenotype.

## Discussion

African populations represent the most genetically diverse populations in the world and this complicates the already inadequate treatment strategies developed for several communicable and non-communicable diseases. Our study in Botswana showed that haplotype composition is diverse between the Bantu-related communities from Serowe/Palapye and Chobe districts, and the San-related communities of the Ghanzi area. This probably reflects the different genetic background and evolutionary history of hunter-gatherers and food producing populations (farming and pastoralists) in Southern Africa^28^. We pooled together the haplotype data from Serowe/Palapye and Chobe districts considering them as a single population since there was no statistical difference in the population structure between the two districts. The frequency for *CYP2B6* 516 G > T is in line with few other studies from Botswana^29,30^. It should be pointed out that looking at the single SNP *CYP2B6* 516 G > T, Serowe/Palapye and Chobe districts do show statistical difference in genotypes distribution (see Table 1). However, haplotypes better predict the population structure than single SNPs^31^, and this observation is also true in *CYP2B6* because of the known linkage among the sites^2^, as confirmed in this study. We identified two haplotypes not yet categorized into alleles that however were already described in other studies^32,33^.

A possible explanation of the difference observed for genotype distribution at 516 polymorphism between the two districts inhabited by Bantu could be due to the HWE of genotypes that was present only in Chobe district. The deviation from the HWE in Serowe/Palapye district was also found for the 983 polymorphism. Deviation from HWE could be due to lack of CC genotypes for *CYP2B6-*983, LD between SNPs at position 516 and 983. In addition, Serowe/Palapye has a wider sample size that increases the likelihood of deviation from the HWE when one of the genotypes (CC) is absent or very rare. Other studies in Africa found absence of 983-CC genotypes which prevented testing for HWE^34,35^. Another possible factor affecting both *CYP2B6*-516 comparisons and HWE in Serowe/Palapye district could be ethnic admixture since it is know that the Bantu-related population of Botswana carries a variable proportion of KhoeSan ancestry^36,37^.

An important result of our study is that the metabolic inferred phenotypes are similar among the three investigated sites. The main reason for this phenomenon could be due to the homeostatic effect of the mutations when taken together. Thus, despite clear and high levels of haplotype diversity among the sites, we observe an inferred phenotypic convergence, with the result that, globally, drug metabolism remains consistent and comparable among the populations studied. It is worth noting that convergent phenotypic evolution is a known phenomenon in biology^38,39^ and also relevant in human populations for several characters included skin pigmentation, lactose tolerance and immune responses^40–43^. The CYP2B6 enzyme plays an important primary role in bioactivating and detoxifying a certain number of procarcinogens and environmental agents^44,45^ as well as processing the arsenal of plant chemical defences introduced with diets^46^. The homogeneous phenotypes among the different sub-samples of this study are probably due to adaptation to the environmental and/or toxicological conditions of the Kalahari and surrounding areas. Based on the Tajima’s D neutrality test results, the two different patterns between Bantu and San could either indicate the presence of balancing selection or alternatively migration or a sudden population contraction in the Bantu population but not in the San. The results could also be possible evidence for positive selection on pre-existing genetic variation. A similar trend was already found by Fuselli *et al*.^47^ for the *CYP2D6* gene and by Podgorná *et al*.^22^ for the *NAT2* gene comparing hunter gatherers with food producing populations in Africa. There is thus some evidence that the current haplotypes might have evolved independently among the different ethnic groups and may thus represent a spectrum in terms of historical and potential adaptations to different ecological and toxicological niches. However, analysis of more genes and control groups will be required to exclude non-evolutionary events (e.g. migration into the Bantu populations) as the underlying cause of the observed results.

According to the metabolic score, expected metabolic phenotypes were obtained: 59.12% of the population study showed a CYP2B6 extensive metabolic phenotype, whereas 36.67% and 4.21% had a reduced and increased metabolic phenotype respectively (see Table 5). The poor and the ultra-rapid metabolic phenotype were observed in 2.1% (n = 12/570) and 0.35% (n = 2/570) of individuals respectively, whereas 34.56% (n = 197/570) showed an intermediate reduced metabolic phenotype, and 3.86% (n = 22/570) had an intermediate increased metabolic phenotype. Individuals with a CYP2B6 reduced metabolic phenotype may have an increased risk of malaria treatment failure when treated with artemisinin derivatives^48^, as well as an impaired outcome of EFV or NVP-based ARTc^10,29^. This effect on malaria therapy efficacy could constitute an important obstacle in the malaria elimination phase, suggesting the need to strengthen the surveillance of AM-LU drug efficacy in Botswana. It is important to note that Chobe is a seasonal malarious area bordering with Namibia, Zambia and Zimbabwe, whereas Serowe/Palapye is more prone to unstable epidemics based on weather patterns^49,50^. Differently, in Ghanzi, which does not usually receive high amounts of rainfall, malaria outbreaks do occur at times, as it is currently the case (http://www.moh.gov.bw/press%20release/MALARIA%20PRESS%20RELEASE.pdf).

Concerning individuals having an inferred ultra-rapid metabolic phenotype, they are rare and the effect of their metabolic pattern seems to affect mainly EFV or NVP-based ARTc by leading to sub-therapeutic drug exposure with a potential increase of risk of selection for viral resistance^10^. Further studies should focus on this fast metaboliser fraction of the population almost neglected in the scientific literature.

Finally, it is worth noticing that drug-drug interaction in malaria and HIV co-infections can lead to therapeutic consequences. For examples, EFV was shown to reduce AM AUC by 80%, tripling the dose of AM needed to compensate EFV-inductive effect^51^. In another study, EFV was shown to reduce LU bioavailability^52^. To ensure antimalarial treatment success in HIV/malaria co-infected patients on EFV-based ART, an increase of dosage or an extension of the duration of AM-LU treatment using the current dose was proposed from several authors^17,53^. Studying how this is affected by drug metabolism phenotype is another important topic that will deserve future attention.

Moreover, malaria infection stimulates HIV replication, causing transient elevation in viral load^54^ that can hamper the management of HIV infected patients, possibly amplifying HIV prevalence^55^ and this could be relevant for Botswana.

Our work has some limitations one being the absence of metabolic phenotypes, since this is a pure genetics study. Furthermore, we did not measure the extent of KhoeSan ancestry, instead we based our definition of San-related ethnicity on family names and sites. However, based on the results from our current study, we can conclude that although ethnically and genetically different, populations in Botswana display convergent evolution in their drug metabolism. The presence of significant numbers of slow and fast metabolisers can significantly impact the emergence and spread of drug resistance in malaria and HIV, either by exposing pathogens to sub-lethal drug doses or inducing non-compliance in patients, with similar consequences. The high frequency of co-infections and the negative interaction between anti-HIV and anti-malaria drugs further exacerbates the risk of resistance emerging. This warrants constant monitoring in the population to identify potential patients with abnormal drug metabolism and adapt treatments accordingly.

## Methods

### Sample collection

The survey was performed from March to May 2012 in Botswana in the broader context of a Malaria Indicator Survey^56,57^. A total of 609 unrelated children aged 2–12 years asymptomatic for malaria were enrolled from primary schools and child welfare clinics: 288 from the Serowe/Palapye district, 159 from the Chobe district and 162 from the Ghanzi (or Gantsi) district. These districts have peculiar ethnic compositions, as shown in previous studies^24,58^. The same protocol for enrollment was followed in all sites. Written informed consent for multiple genetic and epidemiological surveys was obtained from all the subjects’ parents/caregivers before enrollment in the study. This study was conducted in accordance with the guidelines of the Helsinki Declaration of 2000 revised 2013, with the approval from Human Research and Development Division of the Botswana Ministry of Health [PPME-13/18 V (380)] and the Institutional Review Board of the University of Pennsylvania [protocol number 820378]. Three ml of whole blood was collected into EDTA-containing tubes by qualified health care workers.

### DNA extraction and PCR-RFLP conditions

DNA was extracted with Qiagen kits according to the manufacturer’s protocol. *CYP2B6* 516 G > T (rs3745274) detection was carried out using a PCR-RFLP technique according to the protocol of Lavandera *et al*.^59^ with minor modifications. For *CYP2B6* 983 T > C (rs28399499) detection we applied a touchdown PCR-RFLP assay developed in our laboratory^34^. Finally for the purpose of this study we adopted a new in-house protocol for the analysis of the *CYP2B6* 785 A > G (rs2279343) polymorphism. Briefly, we designed two primers that amplify a 223 bp fragment of the *CYP2B6* gene (forward primer: 5′-AACCTGCAGGAAATCAATGC-3′; reverse primer: 5′-CCTTCTTCCCTCCCCATCTTC-3′). For PCR cycling, after 5 min of denaturation at 94 C, the PCR mixture was subjected to the following conditions: 30 s at 94 C, 30 s at 65 C and 30 s at 72 C for 35 cycles, with a final step of 10 min at 72 C. The PCR product was then incubated with NlaIV restriction enzyme that cuts the mutant allele (G) in three fragments of 144 bp, 62 bp and 17 bp; while the wild-type allele (A) is digested in two fragments of 161 bp and 62 bp. The digested fragments were visualized on a 4% agarose gel. Negative control comprised of PCR reaction without a DNA template and controls for human genotyping were utilized after sequencing the different genotypes.

### Hardy-Weinberg Equilibrium evaluation

Evaluation of Hardy-Weinberg equilibrium was performed using the HWSIM software (freely available at http://krunch.med.yale.edu/hwsim/) and Monte-Carlo permutation test performed when genotypic classes had an expected cell size of less than five. Wright’s F statistics was applied to evaluate the expected level of heterozygosity.

### Linkage Disequilibrium, haplotype frequency analysis and Neutrality test

Arlequin v3.5^60^ was used to test for linkage disequilibrium between the three loci and for haplotype reconstruction. Several other studies were able to evaluate haplotype structure and population differentiation looking at functional SNPs in the same gene^61–64^. The input consisted of diploid genotypic DNA data with unknown gametic phase and assuming co-dominance. The expectation-maximization (EM) algorithm used to test for Linkage Disequilibrium (LD) was run for 20,000 permutations and 3 initial conditions, based on recommended criteria. For haplotype reconstruction the EM algorithm was run on the same input file used for the LD test at the haplotype level, with 50 starting points and 1,000 iterations. Other parameters were set to default. The Excoffier–Laval–Balding (ELB) algorithm (with default settings) was used to generate haplotype counts. Population haplotype frequencies were determined both by haplotype counts and estimated by Maximum Likelihood (ML) and were compared using pairwise FSTs calculated using default values.

After determining the gametic phase of the samples using the ELB algorithm in Arlequin, the DnaSP (v6) software^65^ was used to calculate and evaluate the Tajima’s D neutrality test using the total number of mutations.

### Metabolic score

We elaborated and adopted a “metabolic score” to take into account the global effect of the three polymorphisms together, according to an extensive literature linking genetic polymorphisms to functional impact^10,66–68^. For the metabolic score we translated the genotype information into a measure of phenotype using an ‘activity score’ system already adopted for *CYP2D6*^47,69^, for *CYP2A6*^70^ and for *CYP2C19*^71^. The metabolic score adopted is based on the algebraic sum of the individual allele values, according to an additive model for *CYP2B6*^72,73^. The scores were set conferring a −1 value for each slow metabolism allele and +1 for rapid metabolism allele, while an extensive metabolism allele was scored 0. Accordingly, we obtained: 516 GG = 0; 516 GT = −1; 516 TT = −2; 983 TT = 0; 983 TC = −1; 983 CC = −2; 785 AA = 0; 785 AG =  +1; 785 GG =  +2. Each composite genotype was attributed a final metabolic score by summing the single score for each SNP.

To test possible deviation from the normal distribution of the scores the Kolmogorov-Smirnov test was used and the statistic *D* values calculated for each distribution. A related P-value greater than 0.05 indicates normal distribution of data^74^. Bartlett’s test was performed to assess if the assumption of equal variances among the three populations was valid^75^. Comparisons among metabolic scores were calculated using the chi-square statistic.

All the statistical calculations were performed using Statistica 13.0 software (StatSoft).
